# Post-traumatic coxa vara in children following screw fixation of the femoral neck

**DOI:** 10.3109/17453674.2010.501744

**Published:** 2010-07-16

**Authors:** Robert Eberl, Georg Singer, Peter Ferlic, Annelie M Weinberg, Michael E Hoellwarth

**Affiliations:** Department of Paediatric and Adolescent Surgery, Medical University of Graz, GrazAustria

## Abstract

**Background and purpose:**

The rare displaced fractures of the femoral neck in children need accurate reduction and rigid fixation. The implants commonly used for internal fixation in children are pins or screws. We evaluated the long-term outcome in children who sustained fractures of the proximal femur that were treated by screw fixation.

**Patients and methods:**

All 22 children (mean age 12 (5–16) years) with fractures of the femoral neck that were treated with screw fixation (mean 2.4 (1–3) screws) at our department between 1990 and 2006 were evaluated. For measurement of outcome, the Harris hip score (HHS) was used and the development of post-traumatic coxa vara was assessed from the difference in the neck-shaft angle postoperatively and at the latest follow-up examination, after mean 4 (2–15) years.

**Results:**

A loss of reduction was observed in 12 patients. There was a statistically significant correlation between the HHS and the changes in the neck-shaft angle.

**Interpretation:**

Loss of reduction was found in more than half of the children. Screw fixation cannot be recommended for the treatment of femoral neck fractures in children due to a substantial number of post-traumatic coxa vara.

## Introduction

In children, fractures of the proximal femur and femoral neck are rare, representing less than 1% of all pediatric fractures (Chong et al. 1975). Most of these fractures occur due to high-energy trauma. Associations with other considerable soft tissue or skeletal injuries occur in up to one-third of the cases (McDougall 1961). The classification system most commonly used was originally described by Delbet (1907), and popularized by Colonna (1929).

In the child, bone remodeling may realign initially malunited fragments, making accurate anatomic reduction less important in other regions of the body. The physis of the proximal femur is, however, responsible for only 13% of the growth of the thigh and therefore it provides little capacity for spontaneous fracture remodeling (Katz 1983). Thus, displaced fractures of the proximal femur and femoral neck need accurate reduction and rigid fixation. Frequently, the implants used for internal fixation in children are pins or screws (Hughes and Beaty 1994, Morsy 2001, Song et al. 2001).

Complications of this type of fracture are associated with a high long-term morbidity, especially in older children (Hasler and von Laer 2000, Quick and Eastwood 2005), including non-union, leg-length discrepancy, and infection (Canale and Bourland 1977, Davison and Weinstein 1992, Flynn et al. 2002, Moon and Mehlman 2006, Morsy 2001, Ng and Cole 1996). The reported incidence of osteonecrosis, a serious complication, varies between 0% and 92% (Davison and Weinstein 1992, Moon and Mehlman 2006). Moreover, development of post-traumatic coxa vara has been described as a serious complication of fractures of the neck of the femur in both adults and children (Estrada et al. 2002, Morsy 2001, Song et al. 2001, Togrul et al. 2005). However, we could not find any information on the long-term outcome of screw fixation of femoral neck fractures in children in the recent literature and it is unclear whether it provides sufficient fracture stability. We retrospectively evaluated the long-term follow-up of 22 children with fractures of the femoral neck who were treated with screw fixation at our department.

## Patients and methods

A computerized search of the medical records in our department from the previous 16 years (1990 through 2006) was performed to detect all patients with fractures of the femoral neck. Inclusion criteria for our study were operative fracture treatment with screws, a patient's age of younger than 18 years at the time of injury, and open growth plates of the proximal femur and full radiographic records. Of the 40 children identified, 18 were excluded (5 patients with undisplaced and stable Delbet type II and III fractures, 2 patients with a Delbet type I fracture, 7 patients with type IV fracture, and 4 patients with pathological fractures). Mean age at the time of injury in the 22 children (12 girls) included in the study was 12 (5–16) years. 7 fractures were Delbet type II and there were 15 Delbet type III fractures. According to the classification of Pauwels (1935), we identified 4 type I fractures, 16 type II fractures, and 2 type III fractures. 5 patients had sustained additional injuries, 2 of which were ipsilateral. 2 patients had multiple injuries.

All 22 fractures were reduced using an extension table. 12 fractures were stabilized using percutaneous cannulated screws after closed reduction, while in 10 patients open reduction and internal screw fixation was necessary. The diameter of the screws was 4.0 mm in 3 patients and 6.5 mm in the remaining 19 patients. The mean number of implanted screws was 2.4 (1–3). Pauwels type I fractures were stabilized with mean 2.2 screws, type II fractures with mean 2.6 screws, and type III fractures with mean 2.0 screws. No infections occurred. A spica cast was not used in any of our patients. Passive motion exercises started on the second postoperative day, followed by walking with crutches without weight bearing for 6 weeks, after which cycling and swimming was recommended. Contact sports were not allowed for at least 3 months.

At our department, the regular follow-up for dislocated fractures of the femoral neck is until the end of growth, including radiographs of the hip in 2 planes. The latest follow-up examination was done on average 4 (2–15) years after surgery.

The radiographs were analyzed retrospectively by one of the authors who was not the treating surgeon (RE). For characterization of fracture stability, the fracture angle was classified according to the Pauwels classification (types I to III). Conventional follow-up radiographs of the hip were performed in 2 planes. For measurement of the neck-shaft angle on the antero-posterior radiographs, the patella was positioned properly in neutral position concerning rotation. The postoperative neck-shaft angle was compared to that of the unaffected side and to that in the most recent follow-up examination. The angle was evaluated with a first line drawn through the center of the femoral head and through the middle of the narrowest distance of the two cortices of the femoral neck, and a second line following the femoral shaft. To determine the most accurate neck-shaft angle, a CT scan would be necessary to compare the neck-shaft angles to the results obtained from conventional radiographs. The magnitude of exposure to radiation does not, however, justify this diagnostic strategy.

The outcome measurement included the Harris hip score (HHS). In order to reflect the activity of children, the HHS was modified to assess the possibility of participating in sports.

### Statistics

For comparing groups, an ANOVA with Scheffe's test was performed and a p-value of < 0.05 was considered statistically significant.

## Results

**Figure 1.**
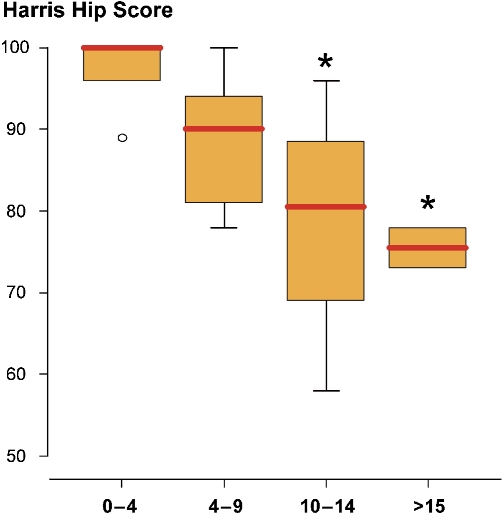
4 categories of changes of the neck-shaft angle plotted against HHS. * indicates p < 0.05 compared to 0–4°.

**Figure 2.**
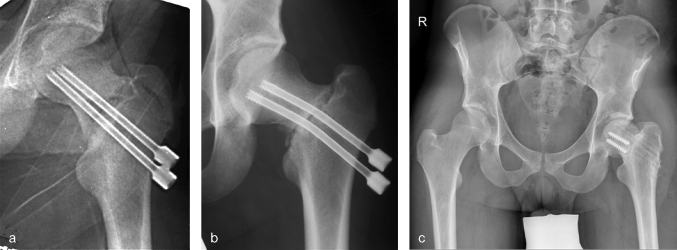
A 16-year-old polytraumatized boy sustained a Delbet type III, Pauwels type III fracture after a motorcycle accident. Fracture reduction was obtained using an extension table. Fracture fixation was performed with two 6.5-mm cannulated screws with open reduction and incision of the joint capsule (a). The axial view detected an area of comminuted fracture. During the course of treatment (tenth postoperative day), implant failure with loss of reduction was observed (b). In a second procedure, the fracture was fixed with 3 solid screws exceeding the growth plate. However, a further loss of reduction was observed 3 weeks after the second procedure. Re-osteosynthesis was done with 3 solid screws with washers. Fracture healing could be achieved with posttraumatic coxa vara, shortening of the femoral neck, and subsequent leg length discrepancy of 2.5 cm at the latest follow-up examination 4.5 years after the injury. Implants remained partially in situ due to breakage of the screws (c).

Loss of fracture reduction (defined as differences in the neck-shaft angle of more than 4 degrees) was found in 12 patients (Table). Patients with differences in neck-shaft angle of between 10° and 14° and more than 15°, respectively, had a lower HHS than patients with an unaltered neck-shaft angle (Figure 1). Post-traumatic coxa vara in 1 child was caused by implant failure (Figure 2).

**Table:** Comparison of the difference of the neck-shaft angle at follow-up examination and Pauwels classification in 22 patients after screw fixation

Difference in neck-shaft angle from the unaffected side (degrees)	n	%	Pauwels classification		
			I	II	III
0–4	10	46	4	6	0
5–9	6	27	0	6	0
10–14	4	18	0	3	1
≥ 15	2	9	0	1	1
Total	22	100	4	16	2

Differences in neck-shaft angle of up to 4 degrees were rated as unaffected

2 patients developed osteonecrosis (1 Ratliff type I and 1 type III). At the time of the most recent visit to the clinic, the patient with the Ratliff type III osteonecrosis (4 years after surgery) had normal radiographs. In the other patient, osteonecrosis of the complete femoral head persisted 7 years after surgery.

## Discussion

Femoral neck fractures in children are associated with a high incidence of complications (Morrissy 1980, Morsy 2001), the most serious, with high long-term morbidity, being osteonecrosis and coxa vara. The reported rates of osteonecrosis vary from 0% to almost 90% (Davison and Weinstein 1992, Moon and Mehlman 2006). In our series, 1 out of 22 patients with a dislocated fracture had persistent radiographic osteonecrosis at follow-up. This low rate of osteonecrosis may be attributed to adequate initial fracture reduction and the timely surgical intervention.

Another serious problem is post-traumatic coxa vara. Lam (1971) found coxa vara to be the most common complication in one-third of patients. Lam patients were treated with a spica cast in non displaced fractures. In dislocated fractures a spica cast was applied after closed reduction or the fracture was reduced with skin or skeletal traction. Operative intervention was performed in patients with futile closed reduction or loss of reduction during the course of treatment.

**Figure 3.**
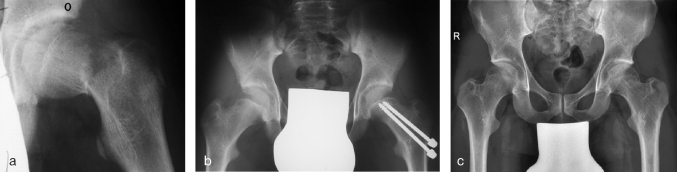
A 14-year-old boy sustained an isolated fracture of the femoral neck type Delbet II, Pauwels II injury with intact medial cortex after a fall from a height (a). Stable fracture fixation could be done with 2 solid 6.5 mm screws following open reduction without exceeding the growth plate. Proper fracture healing was achieved during the course of treatment (b). 13 years after the injury the neck shaft angle was unaffected and the patient had normal functional (c).

Togrul et al. (2005) described the occurrence of coxa vara in half of their non-operatively treated children and considered that many of them should have been operated on (Togrul et al. 2005). Recent reports state that the incidence of coxa vara may be reduced by using internal fixation such as pins or screws (Hughes and Beaty 1994, Morsy 2001, Shrader et al. 2007, Togrul et al. 2005). Interestingly, we found that coxa vara developed despite screw fixation in more than half of the children. Premature physeal closure has been described as one of the etiological factors of coxa vara (Togrul et al. 2005). Incidences of premature physeal closure reported in the literature range from 6% (Ratliff 1962) to 62% (Canale and Bourland 1977). We did not find premature physeal closure in any of our patients, however. Another possible explanation for the high occurrence of coxa vara due to a loss of reduction after accurate initial fracture reduction is implant failure in unstable fractures (Figures 2 and 3). Unintentional early weight bearing and/or high shearing forces in injuries with a fracture line that is more vertical might cause loss of reduction, especially in adolescents (Estrada et al. 2002). As a consequence of our findings, we now use interlocking implants with dimension and profile adapted to children for fixation of fractures with a high index of instability such as vertical fracture line and comminuted fracture. However, at the present time we are unable to confirm this new treatment protocol since data on long-term outcome are still missing.

Screw fixation is an adequate method for treatment of stable fractures and injuries with a fracture line that is more horizontal, with intact medial cortex (Figure 3). We recommend exceeding the growth plate in the case of Delbet type II fractures with short medial fragment, to achieve better fracture stability. Post-traumatic coxa vara with all the resulting long-term complications may be prevented with this treatment protocol.

There are several limitations to our study. Even though we analyzed 22 patients treated over 16 consecutive years, a higher number of patients would be necessary to confirm our results—preferably with a prospective, multicenter study. We measured the outcome with the HHS, which was developed for adult patients with acetabular fractures. To our knowledge, there is no score for evaluation of femoral neck fractures in children and there might be a large ceiling effect in this age group.
